# Immunopathogenesis of Severe Fever with Thrombocytopenia Syndrome: Core Driving Role of Cytokine Storm

**DOI:** 10.3390/cimb48030263

**Published:** 2026-03-01

**Authors:** Yuan Ding, Quanman Hu, Yan Hu, Yanyan Yang, Jundong Chen, Fei Zhao, Saiwei Lu, Li Zhang, Shuaiyin Chen, Guangcai Duan

**Affiliations:** 1 Department of College of Public Health, Zhengzhou University, Zhengzhou 450001, China; yuan082025@163.com (Y.D.); quanmanhu@163.com (Q.H.); zfpenguin@163.com (F.Z.); lsw123985@163.com (S.L.); gcduan@zzu.edu.cn (G.D.); 2 Disease Control and Prevention Center, Xinyang 463600, China; xycdchuy@163.com (Y.H.); jerry12799@163.com (Y.Y.); thelni@163.com (J.C.); zhangli6688@126.com (L.Z.)

**Keywords:** cytokine storm, SFTS virus (SFTSV), immunopathogenesis, multiple organ failure, aberrant immune cell polarization, inflammatory imbalance, targeted immunotherapy, viral immune evasion

## Abstract

Severe fever with thrombocytopenia syndrome (SFTS) is a newly discovered tick-borne disease caused by SFTS virus (SFTSV) infection. Patients present with high fever, thrombocytopenia, and multiple organ dysfunction, with a high mortality rate and a lack of specific treatment, all of which indicate that research on the deterioration mechanism and treatment of this disease is urgent. Currently, multiple studies have indicated that cytokine storm is one of the core factors contributing to the deterioration of the disease. SFTSV inhibits the host’s type I interferon response through its non-structural protein NSs, thereby promoting immune evasion and viral replication. Extensive viral stimulation leads to dysfunction and abnormal polarization of immune cells (including monocytes, macrophages, dendritic cells, T cells, and B cells), triggering the massive release of pro-inflammatory factors(such as interleukin-6 (IL-6), tumor necrosis factor-alpha (TNF-α), and interleukin-1 beta (IL-1β)), anti-inflammatory factors (such as interleukin-10 (IL-10)), and chemokines(such as interferon-gamma inducible protein 10 (IP-10), monocyte chemoattractant protein-1 (MCP-1), and interleukin-8 (IL-8)). This cytokine storm exacerbates the imbalance between pro-inflammatory and anti-inflammatory factors, as well as immune paralysis, leading to vascular endothelial damage, microthrombosis, and ultimately, multi-organ failure, which determines the clinical outcome. Simultaneously, specific cytokines and immune cell phenotypes can serve as biomarkers for disease severity and prognosis. In terms of treatment, this article further summarizes the intervention strategies targeting the aforementioned immune links, including intravenous immunoglobulin (IVIG), tocilizumab (targeting the IL-6 receptor), inhibitors of Janus kinase (JAK) and nuclear factor-kappa B (NF-κB) signaling pathways, interferon, neutralizing antibodies, and other immunotherapy methods. By analyzing the dynamic changes and mechanisms of cytokine storm in the course of SFTS, and summarizing current potential immunotherapy methods, this article aims to provide a theoretical framework for the future treatment of SFTS.

## 1. Introduction

Severe fever with thrombocytopenia syndrome (SFTS) is a novel tick-borne infectious disease caused by the *Dabie bandavirus* (formerly known as SFTS virus, belonging to the family Phenuiviridae, genus *Bandavirus*) [1]. Since its first discovery in China more than a decade ago, SFTS has exhibited significant epidemiological expansion. As of 2023, China has reported a cumulative total of 27,447 cases, and the disease has also been observed in East Asian countries including South Korea, Japan, and Vietnam, with its transmission momentum continuing to intensify [1,2,3]. The high case fatality rate of SFTS (which can reach up to 30% in some areas) [3] coupled with evidence suggesting the emergence of potentially more virulent strains [4] indicates that research into the deterioration mechanisms and treatment of the disease is urgently needed.

The virus is primarily transmitted through the bite of the Asian longhorned tick (*Haemaphysalis longicornis*) [4,5], a species native to eastern Asia that has now invaded multiple countries including the United States, Australia, and New Zealand. (Distribution of *Haemaphysalis longicornis* and associated pathogens: analysis of pooled data from a China field survey and global published data). Upon entering the host’s bloodstream, the glycoprotein Gn on the virus surface specifically binds to the host cell receptor *CCR2*, allowing the virus to enter cells through envelope fusion or endocytosis. Subsequently, the virus releases its segmented single-stranded negative-sense RNA genome in the cells, replicates and transcribes, and synthesizes structural proteins (such as glycoproteins Gn and Gc, as well as nucleoprotein N) and non-structural proteins [1,3]. Among them, non-structural proteins (NSs) have garnered significant research attention due to their close association with severe immune dysregulation, immune evasion, and excessive inflammatory responses after infection.

Clinically, the disease manifests primarily as acute fever, thrombocytopenia, and leukopenia [2,5]. Some patients have mild clinical signs, manifested by high fever, gastrointestinal clinical signs, lymphadenopathy and bleeding manifestations such as petechiae, ecchymosis, or mucosal hemorrhage, and the prognosis is good after active treatment. However, some patients may experience acute exacerbations within 1 to 2 weeks of onset, rapidly progressing to fatal complications such as multiple organ dysfunction syndrome (MODS), disseminated intravascular coagulation (DIC), severe hemorrhage, and encephalopathy, with a high mortality rate [4,6]. Currently, there are no specific antiviral drugs available for SFTS, and clinical management relies mainly on supportive and symptomatic treatments, which have limited efficacy [4,7].

Given the high case fatality rate and the lack of specific treatment for this disease, it is particularly important to study its underlying deterioration mechanisms. In recent years, mounting evidence has indicated that cytokine storm, which refers to the excessive and dysregulated release of pro-inflammatory cytokines and chemokines, plays a pivotal role in the clinical deterioration and prognosis of SFTS, and can also serve as an immune marker to distinguish between mild and severe cases [6,8]. This excessive inflammatory response is not only positively correlated with viral load and mortality [9,10], but more importantly, it is not merely an immune overreaction, but rather a result of the virus’s active immune evasion strategy: on the one hand, it inhibits protective antiviral immunity, and, on the other hand, it amplifies destructive inflammatory cascades, ultimately leading to tissue damage and organ failure [11,12]. Despite ongoing in-depth research, many details remain fragmented and unclear, including the primary sources of cytokines involved in the course of SFTS, their dynamic interactions with different immune cell subsets, and the mechanisms of action in organ damage. These aspects still require further summarization.

This review will systematically integrate existing research evidence to explore the role of cytokine storm in the pathogenesis and deterioration mechanism of severe fever with thrombocytopenia syndrome (SFTS). We will analyze the mechanism underlying the formation of cytokine storm, the interactions between storm-related cytokines and host immune cells, the pathological process of excessive inflammation leading to tissue and organ damage, as well as the potential of cytokine profiles as biomarkers for predicting severe disease. Simultaneously, we will evaluate current and emerging immunotherapy strategies, particularly interventions targeting cytokine storm or its inducing pathways, to provide a theoretical basis for the development of targeted immunotherapy interventions for SFTS.

## 2. The Core Role of Cytokine Storm in the Pathogenesis of SFTS

**Figure 1:**
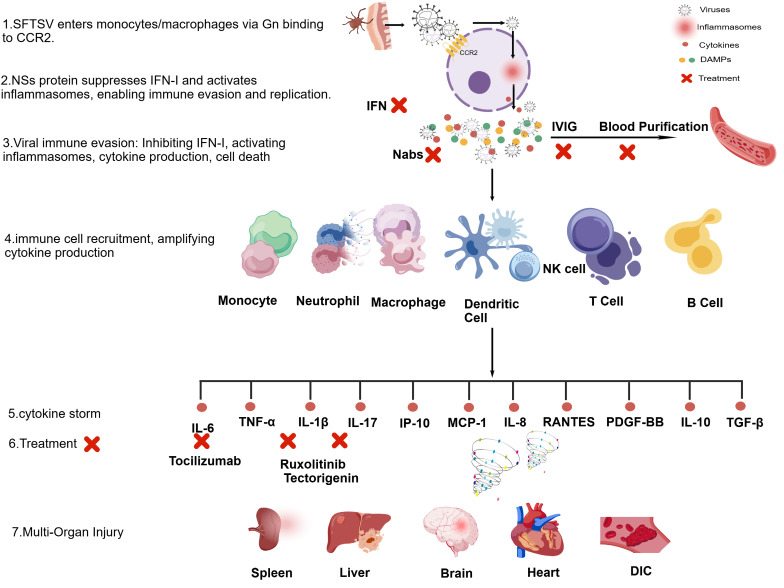
Schematic illustration of the immune disease cascade following SFTSV infection. Symbol Explanations: Solid arrows (→) indicate directional progression or stimulation; red crosses (✖) indicate points of therapeutic intervention; the swirl represents the cytokine storm. Created with biogdp.com.

Cytokine storm (CS), also known as cytokine release syndrome, is a potentially fatal excessive immune response [13,14]. This is manifested as an abnormal and massive release of inflammatory mediators following immune system dysregulation. Specifically, when pathogen-associated molecular patterns (PAMPs) activate the host’s innate immune system, the first wave of cytokines (such as TNF-α, IL-1β, and IL-6) is released. Subsequently, the adaptive immune system is activated, immune cells undergo dysfunction and abnormal polarization, releasing a broader range of cytokines, further amplifying inflammation [15]. In critically ill patients, this inflammatory response is particularly intense. The levels of pro-inflammatory factors continue to rise, creating a strong inflammatory environment, ultimately leading to systemic inflammatory response, vascular endothelial damage, microvascular thrombosis, and multiple organ dysfunction (Figure 1).

Initially, SFTSV invades monocytes and macrophages through the *CCR2* receptor and is recognized by the innate immune system. Subsequently, the virus initiates immune evasion, manifested as the NSs protein inhibiting type I interferon (IFN-I) response and activating the inflammasome, thereby promoting its own replication. A large number of viruses lead to dysfunction and abnormal polarization of various immune cells, including monocytes, neutrophils, macrophages, dendritic cells, NK cells, T cells, and B cells. Simultaneously, cytokines are released in large quantities, resulting in a hyper-inflammatory state, with excessive release of pro-inflammatory and chemotactic cytokines such as IL-6, TNF-α, IL-1β, IL-17, IP-10, MCP-1, and IL-8, as well as dysregulated release of anti-inflammatory cytokines such as IL-10 and TGF-β. This cytokine storm leads to tissue damage and dysfunction in multiple organs, including the spleen, liver, brain, and heart, and promotes disseminated intravascular coagulation (DIC). The figure also summarizes current and potential therapeutic interventions targeting key cytokines (e.g., tocilizumab for IL-6 treatment and ruxolitinib for *JAK/STAT* pathway inhibition to reduce inflammation), as well as broad-spectrum anti-inflammatory strategies such as intravenous immunoglobulin (IVIG) and blood purification. Timely identification of patients with high inflammatory responses through cytokine analysis is crucial for guiding SFTS therapy and improving treatment outcomes. This figure was created by the authors.

Cytokine storm is one of the core factors contributing to the deterioration of various infectious diseases, such as COVID-19 [16] and Ebola hemorrhagic fever [17], and it is also a key factor in the deterioration of patients with severe fever with thrombocytopenia syndrome (SFTS). Animal experiments [18] have shown that mice lacking type I interferon receptors are prone to death after infection, highlighting the importance of interferon in resisting viruses. However, the non-structural protein NSs of SFTSV itself can effectively inhibit interferon production, suggesting that other factors contribute to the death of these mice. Furthermore, these knockout mice showed significantly elevated cytokine levels at the time of death, suggesting that cytokine storm may be one of the important causes of death.

Multiple clinical and immunological studies have found a correlation between cytokine storms and the severity and clinical outcomes of SFTS. In severe patients, serum levels of cytokines and chemokines such as IL-6, IL-10, IP-10 (also known as CXCL10), and MCP-1 are significantly elevated and positively correlated with viral load. These indicators show higher levels and longer duration in fatal cases [8,9]. In contrast, pro-inflammatory factors (such as IL-6 and TNF-α) in mild patients usually decline gradually about one week after onset [19].

Furthermore, some critically ill SFTS patients clinically present with hemophagocytic lymphohistiocytosis (HLH), which is closely related to cytokine storm (CS). HLH itself is a critical syndrome characterized by excessive immune system activation and cytokine storm, further confirming the central role of uncontrolled inflammation in the progression of SFTS [4]. Studies have also shown that the intensity of the cytokine response is closely related to the typical clinical manifestations of SFTS, including the severity of thrombocytopenia, elevated liver enzyme levels, and coagulation abnormalities, suggesting that cytokine storm plays a crucial role in the pathological process of multiple organ damage [14,20,21].

In summary, uncontrolled inflammatory amplification and cytokine storm may be an important immunopathological basis for the severe progression of SFTS, and regulating this process may become an important direction for improving prognosis.

## 3. Dynamic Evolution of Cytokine Storm and Disease Progression

Cytokine storm (CS) in severe fever with thrombocytopenia syndrome (SFTS) is a dynamic process closely related to disease progression. After the incubation period, patients typically experience an early infection phase, an acute phase, and may subsequently enter a recovery phase or develop multiple organ dysfunction. In severe cases, except for the recovery phase, high viral load and excessive inflammatory response coexist in all stages, and both are significantly positively correlated with clinical severity and prognosis [8,22]. The underlying mechanisms are systematically described below in chronological order of the disease, from viral recognition and immune evasion to inflammatory dysregulation.

### 3.1. Viral Recognition and Suppression of the Interferon System

In the early stages of infection, SFTS virus (SFTSV) invades the host and is initially sensed by innate immune cells such as monocytes and macrophages through pattern recognition receptors (PRRs), including *TLR3/7* and *RIG-I*. In a normal immune response, this recognition rapidly activates the *IRF3/7* signaling pathway, subsequently inducing the production of type I interferons (IFN-I) to establish antiviral defense [1,3].

**Figure 2:**
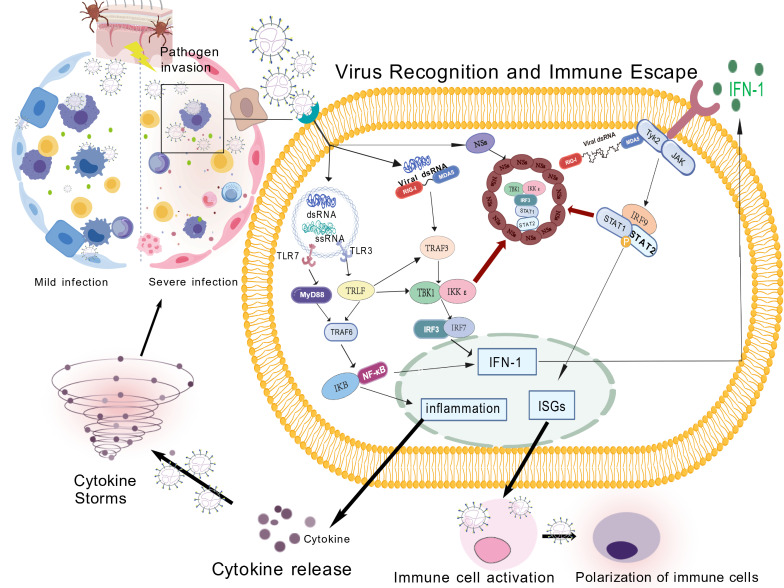
Viral immune recognition and immune evasion. Symbol Explanations: Solid arrows (→) indicate directional progression or stimula-tion; the swirl represents the cytokine storm. Created with biogdp.com.

However, SFTSV has evolved corresponding immune evasion strategies to suppress interferon production [12]: on the one hand, NSs directly binds to and sequesters key molecules of the RIG-I pathway, including *TRIM25* and *TBK1*, inhibiting RIG-I ubiquitination and activation and downstream signaling, thereby suppressing IFN-β transcription [1,2,11]. On the other hand, NSs targets the JAK-STAT pathway, binding to *STAT1* and *STAT2* and sequestering them in inclusion bodies, preventing their phosphorylation, nuclear translocation, and activation of downstream interferon-stimulated genes (ISGs), thus blocking IFN-I and IFN-γ responses at the signal reception level [23,24]. Clinical serological evidence also supports this mechanism: early IFN-β levels are significantly reduced in fatal cases and show a strong negative correlation with viral load [24]; in contrast, IFN-α levels show more complex changes, but its downstream signaling is also inhibited by NSs [25] (Figure 2).

After viral RNA is recognized by *TLR* and *RIG-I* receptors, the body’s *IRF3/IRF7*, NF-κB, and JAK-STAT signaling pathways are activated, inducing the expression of type I interferon (IFN-I), pro-inflammatory factors, and interferon-stimulated genes (ISGs), thereby establishing an antiviral state. However, the NSs protein of SFTSV sequesters signaling molecules, including *MDA5*, *TBK1*, *IRF3*, and *STAT1/2*, into viral inclusion bodies (IBs), thereby inhibiting the IFN-I response to achieve immune evasion. Mildly ill patients establish effective innate immune control, recovering after viral clearance. Severely ill patients, however, experience abnormal immune cell polarization, uncontrolled cytokine release, and pro-inflammatory/anti-inflammatory imbalance due to IFN suppression and persistent high viremia. This ultimately leads to a cytokine storm and a vicious cycle of multi-organ damage. This figure was created by the authors.

### 3.2. Early Infection: Innate Immune Activation and Initial Cytokine Differentiation

Existing research primarily focuses on the peak phase of cytokine storm, with less attention paid to the initial “triggering” stage. By integrating clinical data, we found that certain immune indicators already exhibit significant differences between mild and severe patients in the early stage of infection (or upon admission). We hypothesize that SFTSV, similar to other viral infections [26], may disrupt vascular integrity and evade the host’s antiviral immunity through the early release of abnormal cytokines, thereby promoting systemic virus dissemination. This hypothesis requires further verification in SFTS.

In this stage, monocytes, macrophages, and dendritic cells in the innate immune system are the main targets of SFTSV. In mild cases, these cells release moderate amounts of pro-inflammatory factors such as IL-6 and TNF-α, which help control viral replication. Conversely, in severe cases, the levels of these pro-inflammatory factors are higher in the early stages, and the differences are statistically significant [14,20,22]. Unlike IL-6 and TNF-α, some mediators show a more consistent early pattern across patients of different severity levels, such as the chemokine IP-10 (CXCL10), which is rapidly upregulated in all patients and is responsible for recruiting T lymphocytes to the infection site [19,27,28]. Furthermore, the moderate early increase in IL-1β observed in survivors suggests that when this factor is within a controllable range, it may contribute to viral clearance [29,30,31].

Another key feature of severe cases in the early stages is the premature activation of anti-inflammatory mechanisms. A large release of IL-10 often occurs in the early stages of the disease [32,33], and its increase precedes that of IL-6 [34]. This abnormal early increase in anti-inflammatory cytokines may indicate “immunosuppression” or “immune paralysis.” We speculate that this may be due to a failure of the host’s compensatory response to inflammation. Premature anti-inflammatory feedback may create an immune tolerant environment favorable for continuous viral replication, thereby exacerbating the subsequent cytokine storm and disease progression.

### 3.3. Acute Phase: Mechanisms and Pathological Effects of Cytokine Storm Formation

The acute phase is the core stage of SFTS disease progression, characterized by the viral load reaching its peak. During this stage, SFTSV has already achieved large-scale replication through one of its immune evasion strategies, namely inhibiting interferon production. Simultaneously, the virus employs another immune evasion strategy by hijacking *TBK1* to disrupt the negative feedback regulation of the inflammatory response, leading to pathological overactivation of the NF-κB signaling pathway [24,35], triggering the release of a large number of pro-inflammatory cytokines, accompanied by immune cell dysfunction and abnormal polarization. The resulting “immune cell-cytokine” pathological interaction network creates a self-amplifying vicious cycle, ultimately leading to an irreversible cytokine storm and multi-organ damage.

#### Polarization and Dysfunction of Immune Cells

Monocytes and macrophages, as key components of innate immunity and the main targets of SFTSV infection, exhibit abnormal activation and polarization, which are core features of acute inflammation. On the one hand, after infection, monocyte subtypes transform from classical to intermediate types, the latter showing higher susceptibility to viral infection, accompanied by high expression of interferon-stimulated genes and activation of the complement system, leading to a significant increase in inflammatory cytokine secretion [9,31]. On the other hand, macrophages undergo functional transformation under viral infection stimulation, secreting a large amount of inflammatory mediators such as IL-1β and TNF-α, and are abnormally induced to polarize towards the M2 phenotype. The phagocytic and antiviral functions of M2 macrophages are relatively poor, which may be more conducive to the extensive replication of viruses. Moreover, this type of macrophages exhibits an abnormally increased phagocytosis of platelets, exacerbating thrombocytopenia and coagulation dysfunction [36].

Dendritic cells (DCs) serve as a crucial bridge connecting innate and adaptive immunity, yet their function is severely compromised upon SFTSV infection. In fatal cases, a significant increase in myeloid DC apoptosis has been observed, correlating with reduced expression of cytokines related to differentiation and maturation, such as IL-4 and GM-CSF. The expression of the pattern recognition receptor *TLR3* is also downregulated, leading to impaired maturation and insufficient expression of co-stimulatory molecules [29,37]. Furthermore, plasmacytoid DCs, the main source of type I interferons, have their powerful antiviral interferon production capacity directly or indirectly suppressed by SFTSV [29,30,37]. This impaired DC function may directly lead to the collapse of downstream T cell and humoral immunity.

Neutrophils: Under the strong recruitment of chemokines (such as IL-8 and CXCL1), neutrophils infiltrate infected tissues in large numbers. Although their released neutrophil extracellular traps (NETs) have antiviral effects, in a cytokine storm environment, excessive NETs become key effectors mediating tissue damage. NETs directly damage vascular endothelium, activate the coagulation system to promote microthrombus formation, and interact with platelets to exacerbate coagulopathy, ultimately leading to multi-organ damage in the liver, spleen, and other organs [38,39]. The proportion of low-density neutrophils (LDNs) increases with disease severity, further contributing to the pro-inflammatory environment [40].

NK cells, as a population of innate immune cytotoxic cells, show a significant decrease in number, especially the more cytotoxic *CD16* +*CD56+* subtype, leading to the inability to effectively identify and kill infected cells [41]. Moreover, the virus induces the upregulation of the inhibitory receptor *Siglec-9* on the surface of NK cells, causing activation dysfunction and decreased cytotoxicity [41].

T cells, which should be the main force of cellular immunity, were the most severely damaged, showing a sharp decrease in the number of *CD4*^+^ and *CD8*^+^ T cells. The CD4^+^ T cell subset showed a significant shift, with a decrease in the Th1/Th2 ratio and a significant increase in pro-inflammatory Th17 cells [42,43]. Although activated *CD8*^+^ T cells expressed cytotoxic molecules, they highly expressed inhibitory receptors such as *PD-1* and *Tim-3*, exhibiting a state of functional exhaustion. Furthermore, the abnormal expansion of *CD4*^−^*CD8^−^* double-negative T cells was positively correlated with viral load and coagulation abnormalities, potentially exacerbating inflammation and tissue damage [12,44].

Although the proportion of B cells was significantly increased in fatal cases, their function was abnormal. On the one hand, the antigen-presenting function of B cells was impaired (downregulation of *HLA-DR* and *CD80* expression), preventing effective T cell activation [30,45]. On the other hand, B cells, especially plasma cells, became a potential reservoir for the virus in the blood. Notably, the interferon pathway was suppressed in B cells infected with SFTSV, while these pathways were activated in neighboring uninfected B cells. This “paracrine blockade” may facilitate efficient viral replication within the B cell population [46]. The differentiation of follicular helper T cells (Tfh) was also hindered due to DC dysfunction, leading to disruption of germinal center reactions and failure to produce virus-specific IgG antibodies [47].

### 3.4. Dynamic Changes in Cytokines

**Figure 3:**
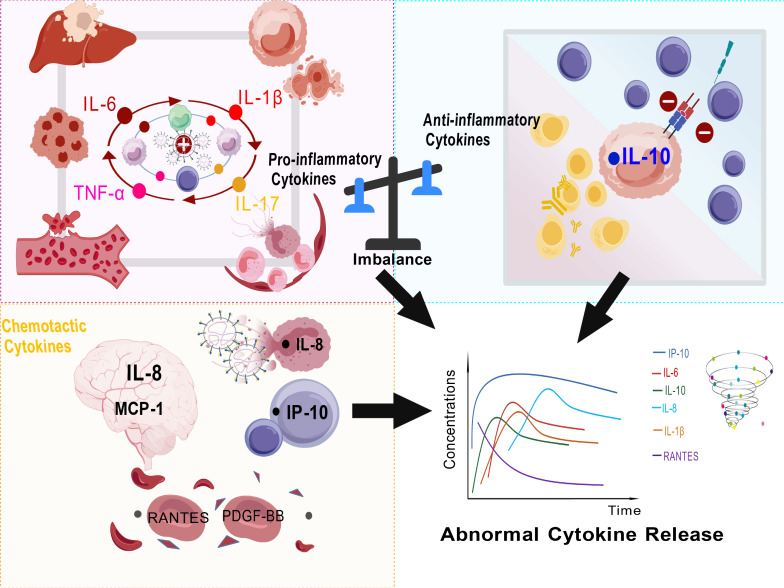
Immunopathogenic mechanisms and dynamic changes in various types of cytokines in SFTS. Symbol explanations: Solid arrows (→) indicate directional progression or stimulation; plus signs (+) indicate exacerbation; minus signs (−) indicate attenuation; the swirl represents the cytokine storm. Created with biogdp.com.

The acute phase is the peak period of the cytokine storm, with a significant increase in various pro-inflammatory factors. Their serum levels are significantly positively correlated with disease severity and prognosis. These factors do not act in isolation but rather form a self-amplifying inflammatory network through mutual reinforcement. The abnormal activation and dysfunction of the aforementioned immune cells are also core driving forces in the formation and maintenance of this network (Figure 3).

The upper left image illustrates the generation of a high-inflammatory environment. A large and sustained number of viruses promote the continuous production of pro-inflammatory cytokines (such as TNF-α, IL-6, IL-1β, IL-17), which mutually amplify each other, leading to extensive damage to cells and tissues (macrophages, liver, vascular endothelium). The upper right image depicts the contradiction between pro-inflammatory and anti-inflammatory responses. The early release of a large amount of anti-inflammatory cytokine IL-10 can, on one hand, inhibit MHC class II expression on macrophages and reduce antigen presentation, thereby limiting excessive pro-inflammatory responses; on the other hand, it promotes B-cell differentiation into plasma cells and antibody production. These contradictions may lead to immune paralysis. The lower left image shows the chemotactic effect of chemokines. For example, IP-10 recruits T cells, and as cerebrospinal fluid MCP-1 (*CCL2*) and IL-8 (*CXCL8*) increase, neutrophils are recruited to the site. The lower right image illustrates the dynamic changes in cytokines in severe patients. Various cytokines (such as IL-6, IL-10, IP-10, MCP-1, IL-8, IL-1β, RANTES) exhibit different concentration trajectories over time. This figure was created by the authors.

#### 3.4.1. Pro-Inflammatory Cytokines

IL-6 mainly originates from activated monocytes and macrophages [48], and in severely ill patients, it is driven by high viral load. Its plasma concentration can reach 4–5 times that of mildly ill patients and remains at high level [19]. IL-6 activates the JAK-STAT pathway, forming a positive feedback loop that further promotes the secretion of itself, TNF-α, and IL-1β [48]. Clinically, excessive IL-6 expression is closely associated with thrombocytopenia and liver damage, possibly through enhanced platelet activation and promotion of inflammatory cell infiltration in the liver [8,48]. In addition, IL-6 synergizes with TGF-β to drive the differentiation of *CD4*^+^ T cells into Th17 cells, thereby increasing IL-17 production and exacerbating inflammation [42,48]. Th17 polarization is one of the typical immune characteristics of severe SFTS, directly contributing to the continuous deterioration of the inflammatory environment [42].

TNF-α is mainly secreted by activated macrophages and T cells, and shows persistently high expression in severely ill patients [2]. It induces the transcription of various pro-inflammatory genes by activating the NF-κB and MAPK pathways. More importantly, TNF-α can directly induce apoptosis: binding to *TNFR1* activates the caspase cascade reaction, which is particularly significant in the liver, leading to massive hepatocyte death and liver failure [14,49]. TNF-α also synergizes with IL-6 and IFN-γ to promote leukocyte adhesion to endothelial cells, aggravating microvascular leakage, thrombosis, and tissue damage. The positive feedback loop between TNF-α and IL-6 is one of the key mechanisms for the escalation of the cytokine storm [14].

The maturation and release of IL-1β depend on *NLRP3* inflammasome activation. SFTSV can activate *NLRP3* through various pathways, including viral RNA, mitochondrial ROS production, and potassium ion efflux, followed by *caspase-1* cleavage of the precursor form into active IL-1β [50]. Infected macrophages are the main source, and massive pyroptosis-like release further amplifies the local pro-inflammatory environment. Although moderately elevated IL-1β in the later stages may contribute to antiviral defense, abnormally high expression in the acute phase undoubtedly accelerates inflammation spread and organ damage [29,31].

IL-17, primarily secreted by Th17 cells, is significantly elevated in the late acute phase [42]. It mainly acts on epithelial and endothelial cells, inducing the expression of chemokines such as IL-8, *CXCL1*, and *CXCL2*, thereby strongly recruiting and activating neutrophils. The accumulated neutrophils release toxic substances such as reactive oxygen species and elastase, directly causing tissue damage. IL-17 synergizes with TNF-α to amplify inflammation and promotes IL-6 production through positive feedback, forming another amplification loop [42,43]. Th17 expansion and the resulting surge in IL-17 are directly related to *CD4*^+^ T cell dysfunction in critically ill patients [42].

In addition to the core factors mentioned above, other pro-inflammatory factors such as IL-1α, IL-18, and LIF are also persistently and significantly higher in non-survivors compared to survivors, further reinforcing uncontrolled inflammation [10,51].

#### 3.4.2. Chemokine Network Remodeling and Immune Cell Recruitment

The chemokine network is primarily responsible for the directed recruitment of immune cells to sites of infection or injury. In SFTS, it exhibits a distinct polarization: most chemokines are upregulated, promoting inflammation amplification, while a few are selectively downregulated, reflecting immunosuppression and delaying viral clearance.

Significantly upregulated chemokines:

IP-10 (*CXCL10*): Levels are significantly elevated and highly correlated with viral load and disease severity. It primarily recruits activated T cells and monocytes to the inflammatory site [27,32]. High IP-10 is often accompanied by a decrease in peripheral blood NK cells and is a commonly used prognostic indicator [52].

MCP-1 (*CCL2*): Significantly elevated in severe patients, its increased concentration in cerebrospinal fluid is closely associated with SFTS-related encephalopathy/encephalitis, possibly promoting viral replication in the brain by disrupting the blood–brain barrier [53,54]. MCP-1 remains higher than normal during the recovery phase, suggesting residual inflammation [51].

IL-8 (*CXCL8*): Significantly elevated in severe cases, as a potent neutrophil chemoattractant, it drives a large number of neutrophils to infiltrate the skin and organs, exacerbating local damage and promoting NET formation [55]. Excessive NETs damage the endothelium, activate coagulation, and worsen consumptive coagulopathy, which is a key link in multiple organ damage [38,39].

Other key members: MCP-3 (*CCL7*), *CCL20* (MIP-3α), *CCL3* (MIP-1α), and *CCL4* (MIP-1β) are rapidly/persistently elevated in non-survivors or high-risk groups, correlating with the risk of death and serving as potential prognostic markers [33].

Selectively downregulated chemokines: Contrary to the upregulation of most chemokines, RANTES (*CCL5*) and PDGF-BB expression is significantly reduced in SFTS patients [8,19]. Thrombocytopenia leads to the depletion of their storage pool. Reduced RANTES may weaken T cell and monocyte recruitment, delaying viral clearance [56]; reduced PDGF-BB affects vascular repair and remodeling, exacerbating bleeding tendencies [57].

#### 3.4.3. Anti-Inflammatory Cytokines

Anti-inflammatory factors exhibit a dual role in SFTS: they may initially attempt to limit excessive inflammation, but their abnormal expression often exacerbates immunosuppression and viral persistence.

The role of IL-10 in SFTS is complex. On the one hand, it can inhibit the expression of *HLA-DR* and *CD86* on the surface of macrophages and reduce the secretion of pro-inflammatory mediators such as TNF-α and IL-6, thus limiting excessive inflammation [30,32]. However, more importantly, the pathological effects of IL-10 are actively utilized and amplified by the virus in SFTSV infection. The viral non-structural protein NSs interacts with the host protein *ABIN2*, activating the *TPL2* kinase signaling pathway, thereby specifically upregulating the expression of IL-10 [23]. Simultaneously, NSs also induces microRNA miR-146b, further promoting IL-10 production and driving macrophage polarization towards the M2 phenotype, actively creating an immunosuppressive microenvironment [9,32]. This series of changes not only weakens the antiviral function of macrophages but may also indirectly inhibit the activation of *CD4*^+^ T cells. Although IL-10 has the potential to promote B-cell differentiation, humoral immunity is still severely impaired (absence of specific IgG, failure of antibody class switching, plasma cell deficiency), indicating that IL-10-mediated regulation fails to reverse immune collapse and may even exacerbate immune tolerance [30,33,45].

TGF-β shows significant temporal and prognostic differences in the acute phase: levels are significantly lower in the fatal group than in the non-fatal group in the early stages, while in recovering severe patients, levels increase significantly multiple times during the course of the disease, maintaining extremely high levels throughout [5,33,34]. Low expression in the early stages leads to difficulty in controlling inflammation, while high expression in the later stages participates in inflammation resolution and tissue repair, with its decline occurring later than that of IL-10. Changes in TGF-β levels are closely related to prognosis and are important dynamic biomarkers [5,33]. In summary, during the acute phase of SFTS, the cytokine storm is essentially a self-amplifying vicious cycle driven by a high viral load, maintained by multi-level dysfunction of immune cells and an imbalance in the pro-inflammatory/anti-inflammatory cytokine network. This ultimately leads to uncontrolled systemic inflammation, vascular endothelial damage, microthrombus formation, and substantial damage to multiple organs, determining the rapid progression of the disease towards a fatal outcome.

## 4. Recovery Period and Fatal Outcomes: The Resolution or Persistence of Cytokine Storm and Its Impact on Systemic Organ Damage

After the acute phase, the prognosis of patients rapidly diverges into two distinct trajectories: some patients experience a gradual weakening of the cytokine storm and a gradual restoration of immune homeostasis while others develop highly fatal complications within a short period of time, accompanied by an intensification of the cytokine storm, ultimately leading to irreversible multi-organ damage or fatal outcomes [58].

### 4.1. Characteristics of Severely Ill or Fatal Patients

This section will elaborate from two levels: firstly, analyzing the “common pathway” leading to involvement of multiple organs throughout the body, namely systemic vascular-coagulopathy; subsequently, exploring the “specific pathway” where different organs exhibit specific clinical manifestations.

#### 4.1.1. Common Pathway: Systemic Vascular-Coagulopathy Driven by Cytokine Storm

In patients with SFTS who have a poor prognosis or die, the cytokine storm does not subside but continues to amplify through multiple mutually reinforcing pathways, leading to extensive vascular-coagulopathy-organ damage, which is the common basis of multi-organ injury:

Endothelial damage and vascular leakage: SFTSV can directly infect endothelial cells, or activate mast cells to release bioactive mediators such as chymase and tryptase, thereby increasing endothelial cell permeability and inducing vascular leakage [59]. This change in permeability begins to emerge from the 4th to 5th day after infection, during which the levels of pro-inflammatory cytokines (IL-6, MCP-1, TNFα, IFNγ, RANTES, IL-1β) in serum and tissues reach their peak, suggesting that cytokine storm may be one of the factors exacerbating vascular leakage [60].

Immune thrombosis: Pro-inflammatory factors such as TNF-α, IL-1β, and IL-6 are the core mediators driving immunothrombosis [2,14]. Among them, IL-1β is matured and released through the activation of inflammasomes (e.g., *NLRP3*, *NLRC4*, *AIM2*). The activated inflammasomes also mediate the release of tissue factor via *caspase-1*, accompanied by the formation of *GSDMD* pores. These factors promote the outflow of intracellular procoagulant substances, thereby inducing the formation of neutrophil extracellular traps (NETs) [33,50,61]. NETs and tissue factor together form the scaffold of immunothrombosis, initiating the coagulation cascade [39,61]. At the same time, a positive feedback loop exists between IL-1β, IL-6, and TNF-α, mutually promoting their production and synergistically transforming the vascular endothelium from an anticoagulant phenotype to a procoagulant phenotype, increasing vascular permeability.

Thrombocytopenia and worsening of coagulation disorders: SFTSV can directly infect platelets, inducing pyroptosis or apoptosis. Under the influence of abnormally activated macrophages (such as those polarized to M2 by the *STAT3/miR-146b* axis), the phagocytosis of self-platelets and blood cells is enhanced, further exacerbating thrombocytopenia and coagulation factor consumption [36]. Simultaneously, the excessive formation of NETs directly damages microvessels, promoting the widespread formation of microthrombi, ultimately leading to disseminated intravascular coagulation (DIC) [39,61], laying the foundation for systemic organ ischemic damage.

#### 4.1.2. Specific Pathways: Organ-Specific Injury Mechanisms Mediated by Cytokine Storm

Based on the common pathway, different organs have specific injury mechanisms, leading to heterogeneity in clinical manifestations:

Central nervous system injury is one of the important lethal factors in severe SFTS, and its occurrence is closely related to the imbalance of chemokines driven by cytokine storm. Chemokines such as MCP-1 and IL-8 continue to increase, and significantly increase in cerebrospinal fluid, which may be related to the disruption of the blood–brain barrier. High levels of chemokines promote the migration of monocytes and neutrophils to the central nervous system, exacerbate local inflammatory reactions, and lead to severe neurological complications [28,53,54].

Respiratory System: High concentrations of inflammatory cytokines and NETs in circulation may damage alveolar capillaries, leading to acute respiratory distress syndrome (ARDS), characterized by progressive dyspnea and hypoxemia [13,62].

Liver and Other Parenchymal Organ Damage: The synergistic effect of pro-inflammatory cytokines (e.g., IL-6, TNF-α) and immune cell infiltration (e.g., neutrophils, monocytes) can induce hepatocyte apoptosis and liver inflammatory damage, which is a common feature in fatal cases [8,14,49,63]. Similarly, other organs (such as the kidneys and myocardium) also suffer secondary damage due to systemic inflammation and microcirculatory disturbances.

### 4.2. Characteristics of the Recovery Phase

In contrast, in patients with mild disease or those who successfully enter the recovery phase, the viral load decreases to below the detection limit around 14 days after symptom onset [22,64]. Levels of pro-inflammatory factors (IL-6, IL-1β, TNF-α) and key chemokines (IL-8, *CXCL10*) gradually decrease, and the anti-inflammatory factor IL-10 also decreases simultaneously, leading to a gradual resolution of the immune response. Immune cell function recovers, the inflammatory network is deactivated, preventing further organ damage, and ultimately leading to clinical recovery.

## 5. Immunotherapeutic Strategies: Advances in Drug Development and Prospects for Clinical Application

### 5.1. Current Status and Limitations of Standard Treatments for Severe Fever with Thrombocytopenia Syndrome (SFTS)

At present, the clinical management of SFTS is mainly symptomatic supportive treatment, and the existing standard treatment mainly includes two types of strategies: antiviral and anti-inflammatory: In antiviral, Ribavirin has been shown to inhibit SFTSV replication in vitro experiments [65], and Favipiravir (T-705) has shown antiviral potential in preclinical studies [66]. In terms of anti-inflammatory, glucocorticoids (such as methylprednisolone, dexamethasone) can quickly inhibit systemic inflammation and stabilize vascular endothelium, which is of certain value to critically ill patients with neurological clinical signs or shock [67,68]. Therapeutic plasmapheresis (TPE) can remove inflammatory factors and viruses from the circulation and play an important role in the rescue of critically ill patients [69,70].

However, the above treatment methods still have certain limitations: ribavirin has not significantly reduced the mortality rate of patients in clinical studies [22], and the efficacy of favipiravir in critically ill patients needs more evidence to support it [66]; improper use of glucocorticoids may inhibit T cell function, increase the risk of infection, and may delay viral clearance with early high-dose use [71,72]; TPE operation is invasive and costly, and has risks of complications, making it difficult to promote at the grassroots level [72]. Therefore, there is an urgent need for more specific intervention strategies for SFTS treatment that target key immunopathological links.

### 5.2. Core Immunotherapeutic Agents: Precision Interventions Targeting Pathological Core

#### 5.2.1. Intravenous Immunoglobulin (IVIG): Modulating Immune Homeostasis

Intravenous immunoglobulin (IVIG) is derived from the pooled plasma of healthy individuals and is rich in a broad spectrum of IgG antibodies, exhibiting both anti-inflammatory and immunomodulatory properties. IVIG functions by inhibiting excessive inflammatory responses, regulating the activities of monocytes/macrophages and T cells, thereby improving the state of “immune paralysis” [73]. Clinical studies indicate that IVIG, whether used alone or in combination with other therapies, can help reduce mortality rates in critically ill patients and amelate complications. Higher total doses and prolonged treatment duration may further optimize patient outcomes [74]. Although IVIG does not contain specific neutralizing antibodies against SFTSV, it can exert therapeutic effects by suppressing cytokine storms, making it particularly suitable for immunomod in patients with mild to moderate disease [75,76]. Nevertheless, further prospective studies are required to establish the optimal dosage and appropriate patient populations for its use.

#### 5.2.2. Anti-IL-6 Pathway Agents (Tocilizumab): Targeting the Core of the Inflammatory Storm

Interleukin-6 (IL-6) is a key pro-inflammatory mediator in the cytokine storm associated with SFTS. Tocilizumab, a humanized monoclonal antibody targeting the *IL-6* receptor, specifically blocks the activation of the IL-6 signaling pathway and offers several advantages: it does not interfere with the body’s antiviral immune responses, thereby avoiding the widespread immunosuppressive effects of glucocorticoids on T cells [71,77]. Clinical studies have shown that patients receiving tocilizumab exhibited lower mortality rates at both 14 and 28 days compared to the therapeutic plasma exchange (TPE) group, with equivalent reductions in viral load [78,79]. Furthermore, tocilizumab demonstrates good safety profiles and superior cost-effectiveness. When used in combination with glucocorticoids, it can further synergistically enhance anti-inflammatory effects, making it particularly suitable for patients experiencing moderate to severe inflammatory states [80].

#### 5.2.3. Inflammatory Signaling Pathway Inhibitors (Ruxolitinib, Tectorigenin): Novel Strategies for Upstream Intervention

Ruxolitinib, a *JAK1/2* inhibitor, targets and suppresses key inflammatory signaling pathways, including those mediated by IFN-γ, and blocks the abnormal activation of NF-κB and MAPK pathways. Clinical reports indicate that its application in patients with high inflammatory states can promote clinical recovery without severe adverse effects [81]. When used conjunction with standard treatments, ruxolitinib significantly improves 28-day survival rates reduces ICU admission rates, and accelerates platelet recovery as well as the alleviation of neurological symptoms [82].

Tectorigenin, a natural NF-κB inhibitor, can inhibit inflammation initiation mediated by *TLR-7* and reduce the transcription and release of inflammatory factors such as IL-6 and TNF-α. In *IFNAR^−^/^−^* mouse models, this compound significantly alleviates inflammatory cell infiltration and tissue damage, demonstrating its potential as an adjunctive therapy for SFTS. However, further research is needed to validate its clinical translational value [83].

#### 5.2.4. Interferons (IFN): Early Intervention to Compensate for Immunodeficiency

In response to the mechanism by which the SFTSV NSs protein inhibits the host interferon response, early supplementation with exogenous interferon serves a dual purpose: it activates the host antiviral signaling pathways to compensate for innate immune deficiencies and directly inhibits viral replication. Research indicates that the combination of interferon with ribavirin can achieve significant viral suppression even at low doses, without increasing the risk of immunosuppression. This combination is particularly applicable for early intervention in patients with mild to moderate disease [84].

#### 5.2.5. Neutralizing Antibodies (Nabs): A New Direction for Specific Antiviral Immunotherapy

Neutralizing antibodies are specific immunoglobulins (primarily IgG) produced by the body in response to SFTSV infection, and they can also be artificially generated using techniques such as genetic engineering and mRNA technology. The core mechanism of these antibodies lies in their precise targeting of the SFTSV surface glycoproteins *Gn*, which blocks the virus from binding to host cells and subsequent replication, thereby halting the infection at its source [85]. At the same time, it can indirectly inhibit cytokine storms, reduce multi-organ damage, and achieve the dual effect of “antiviral + anti-inflammatory” [85,86]. Preclinical studies have confirmed that human monoclonal neutralizing antibodies, especially through combinations of antibodies targeting different epitopes, completely protect mice from lethal attack by SFTSV [85,87]. However, the clinical translation of neutralizing antibodies still faces challenges, such as the risk of viral escape mutations and high production costs. The optimal dosing regimen, timing of treatment, and combination therapy strategies need to be further validated through multi-center clinical trials.

The core advantage of immunotherapy lies in its ability to precisely target the immunopathological aspects of SFTS. Given the coexistence and mutual promotion of high viral load and persistent inflammation in SFTS, early intervention may prioritize targeting high viral load as the “master switch”, especially by inhibiting IFN-I through exogenous IFN supplementation and antiviral drugs, to prevent downstream inflammatory amplification caused by excessive activation of NF-κB [7]. If inflammation intensifies independently, treatment should shift to targeted inhibition of amplification pathways, such as *IL-6R* blockade (tocilizumab), *JAK* inhibition (ruxolitinib), or NF-κB inhibitors, to control cytokine storms [88]. For some severe patients with high concentrations of inflammatory factors or viral components in the blood, adjuvant TPE or blood purification may be considered to rapidly eliminate these factors. Therefore, a stratified treatment strategy may be more suitable for the clinical management of SFTS patients, including: (1) early febrile phase: preferentially using antiviral drugs (such as Fapilavir) combined with IFN to reduce viral load and enhance innate immunity; (2) acute phase and progression to multiple organ dysfunction (MOD) phase: adding targeted anti-inflammatory drugs such as tocilizumab or ruxolitinib to control cytokine storms, possibly combined with intravenous immunoglobulin (IVIG) for immune modulation; (3) severe/critical cases: incorporating supportive measures such as plasma exchange (TPE) or glucocorticoids as salvage therapy, while monitoring for complications.

## 6. Conclusions

In summary, cytokine storm plays a central role in the pathogenesis of SFTS, driven by a dynamic process involving viral immune evasion, host immune system dysregulation, and an imbalance between pro-inflammatory and anti-inflammatory networks. This process leads to widespread vascular endothelial damage, coagulation dysfunction, and multi-organ failure. Cytokine storm is also a key factor in predicting disease progression and prognosis. Current clinical strategies primarily focused on supportive treatment have limited efficacy, while novel immunotherapies such as targeting cytokine pathways (e.g., anti-IL-6 therapy), modulating immune homeostasis (e.g., IVIG), and applying neutralizing antibodies show potential in intervening in this pathological process.

However, the complexity of cytokine storms—including the dynamic changes in their cellular origins, key regulatory nodes at different stages of the disease process, and the spatiotemporal evolution of pro-inflammatory and anti-inflammatory networks—still needs to be further elucidated. Future research can start from the following operational paths: firstly, utilize single-cell and spatial transcriptomics to precisely locate the main cytokine-producing cells and their tissue distribution; secondly, construct a dynamic evolution model of the cytokine network through longitudinal multi-omics cohort analysis to identify key regulatory nodes; finally, conduct time-point-specific intervention experiments in animal models to verify the functional causality of these nodes, thereby clarifying the optimal intervention window and priority targets. These strategies are expected to fill knowledge gaps and promote the transformation of SFTS from empirical supportive treatment to mechanism-oriented, temporally precise immune intervention.

## Data Availability

No new data were created or analyzed in this study.
